# Impact of a delirium prevention project among older hospitalized patients who underwent orthopedic surgery: a retrospective cohort study

**DOI:** 10.1186/s12877-019-1303-z

**Published:** 2019-10-26

**Authors:** Jung-Yeon Choi, Kwang-il Kim, Min-gu Kang, Young-Kyun Lee, Kyung-Hoi Koo, Joo Han Oh, Young Ho Park, Jeewon Suh, Nak-Hyun Kim, Hyun-Jung Yoo, Jahyun Koo, Hyun Mi Moon, Eun Hui Kim, Kayoung Park, Cheol-Ho Kim

**Affiliations:** 1 Department of Internal Medicine, Seoul National University Bundang Hospital, Gumi-ro 166, Bundang-gu, Seongnam-si, Kyeongi-do 13620 Republic of Korea; 2 Department of Internal Medicine, Seoul National University College of Medicine, 103 Daehak-ro, Jongno-gu, Seoul, 03080 Republic of Korea; 3 Department of Internal Medicine, Chonnam National University Bitgoeul Hospital, 80, Deongnam-gil, Nam-gu, Gwangju, 61748 Republic of Korea; 4 Department of Orthopedic Surgery, Seoul National University Bundang Hospital, Gumi-ro 166, Bundang-gu, Seongnam-si, Kyeongi-do 13620 Republic of Korea; 5 Department of Orthopedic Surgery, Seoul National University College of Medicine, 103 Daehak-ro, Jongno-gu, Seoul, 03080 Republic of Korea; 6 Department of Neurology, Seoul National University Bundang Hospital, Gumi-ro 166, Bundang-gu, Seongnam-si, Kyeongi-do 13620 Republic of Korea; 7 Department of Nursing, Seoul National University Bundang Hospital, Gumi-ro 166, Bundang-gu, Seongnam-si, Kyeongi-do 13620 Republic of Korea; 8 Department of Pharmacy, Seoul National University Bundang Hospital, Gumi-ro 166, Bundang-gu, Seongnam-si, Kyeongi-do 13620 Republic of Korea

**Keywords:** Delirium, Quality improvement project, Multidisciplinary geriatric intervention

## Abstract

**Background:**

Postoperative delirium (POD) is a common clinical syndrome with significant negative outcomes. Thus, we aimed to evaluate the feasibility and effectiveness of a delirium screening tool and multidisciplinary delirium prevention project.

**Methods:**

A retrospective cohort study was conducted at a single teaching center in Korea. A cohort of patients who underwent a delirium prevention program using a simple delirium screening tool from December 2018 to February 2019 (intervention group, *N* = 275) was compared with the cohort from the year before implementation of the delirium prevention program (December 2017 to February 2018) (control group, *N* = 274). Patients aged ≥65 years who were admitted to orthopedic wards and underwent surgery were included. The incidence rates of delirium before and after implementation of the delirium prevention program, effectiveness of the delirium screening tool, change in the knowledge score of nurses, and length of hospital stay were assessed.

**Results:**

The sensitivity and specificity of the screening tool for the incidence of POD were 94.1 and 72.7%, respectively. The incidence rates of POD were 10.2% (control group) and 6.2% (intervention group). The odds ratio for the risk reduction effect of the project related to the incidence of POD was 0.316 (95% confidence interval: 0.125–0.800, *p* = 0.015) after adjustment for possible confounders. The delirium knowledge test score increased from 40.52 to 43.24 out of 49 total points (*p* < 0.001). The median length of hospital stay in the intervention and control groups was 6.0 (interquartile range, 4–9) and 7.0 (interquartile range, 4–10) days, respectively (*p* = 0.062).

**Conclusion:**

The screening tool successfully identified patients at a high risk of POD at admission. The POD prevention project was feasible to implement, effective in preventing delirium, and improved knowledge regarding delirium among the medical staff.

**Trial registration:**

None.

## Background

Postoperative delirium (POD) is a common clinical syndrome characterized by inattention, fluctuating levels of consciousness, and disorganized thinking. Significant negative consequences associated with POD include falls, immobilization, institutionalization, prolonged hospital stay, mortality, and increased healthcare expenses [1, 2]. The incidence of POD has been reported to vary, depending on the diagnostic tool used or the type of surgery performed. Orthopedic surgery, especially hip fracture surgery, has the highest incidence of POD, most likely owing to the urgent insult and complex comorbidity among these patients [3, 4].

Delirium may be due to multiple modifiable risk factors such as dehydration, urinary retention, medication, and malnutrition, as well as unmodifiable risk factors such as advanced age and pre-existing cognitive impairment. As such, between 30 and 40% of delirium cases are known to be preventable [5, 6]. Although several studies on the effects of pharmacological interventions in preventing delirium have been conducted, which have focused on agents such as antipsychotics, acetylcholinesterase inhibitors, melatonin, alpha-2 agonist, benzodiazepine, steroid therapy, statins, and gabapentin, results regarding the efficacy of any one particular pharmacological agent are inconsistent and conflicting [7].

The Hospital Elder Life Program (HELP) was the first evidence-based approach aimed to prevent delirium that described a multicomponent intervention for potentially modifiable clinical risk factors [5]. HELP provided multiple strategies for addressing modifiable risk factors such as mobilization, orientation, sensory adaptation, social interaction, non-pharmacological intervention for sleep and anxiety, and assistance with meals and hydration. The implementation of HELP was reported to successfully reduce incidence of delirium and length of hospital stay and prevent patient functional decline and readmission, and it was shown to be cost-effective. However, the program requires an interdisciplinary core team that includes a certified nurse specialist, a geriatrician, and trained volunteers [8–11]. Due to limited adequate compensation systems and the need to ensure a sufficient number of volunteers, it was reported that most hospitals were unable to implement the delirium prevention programs or that the protocols were inconsistently implemented with variable adherence [12, 13]. Accordingly, developing a screening tool to identify high-risk patient groups who might experience delirium in the future and a methodology for a delirium prevention strategy that can be performed with a smaller number of healthcare providers and caregivers is necessary.

In this study, we aimed to evaluate 1) the performance of a simple POD screening tool to correctly identify individuals at a higher risk of developing POD, 2) whether a quality improvement (QI) project that included education, screening, and a multicomponent preventive strategy would improve outcomes, and 3) whether the medical staff’s degree of knowledge about delirium improved from pre- to post-education.

## Methods

### Study design and setting

This study, which used a before-after approach to evaluate the effectiveness of a project designed to prevent POD, was conducted at Seoul National University Bundang Hospital, a 1300-bed teaching hospital. We defined two study phases, considering annual variation in the characteristics of orthopedic surgical patients, as follows: 1) an evidence-based delirium prevention project phase conducted between December 2018 and February 2019 (intervention group) and 2) a phase involving patients admitted to the same wards during the same time period 1 year earlier, from December 2017 to February 2018 (control group). All inpatients aged ≥65 years who underwent orthopedic surgery and had been admitted to either of the two orthopedic wards (ward 61 and 62) from both the intervention and control study phases were included. Patients who were delirious at the time of hospital admission were excluded from the study. To identify the effect of the delirium prevention protocol application in the general ward, we analyzed the data of all patients in both the intervention and control groups, although some patients missed screening or intervention for reasons such as an examination or scheduled surgery in the intervention group.

The study protocol was reviewed and approved by the Seoul National University Bundang Hospital Institutional Review Board, and the requirement for informed consent was waived (IRB No. B-1904/534–104).

### A simple screening tool to identify patients at risk of POD

**Fig. 1:**
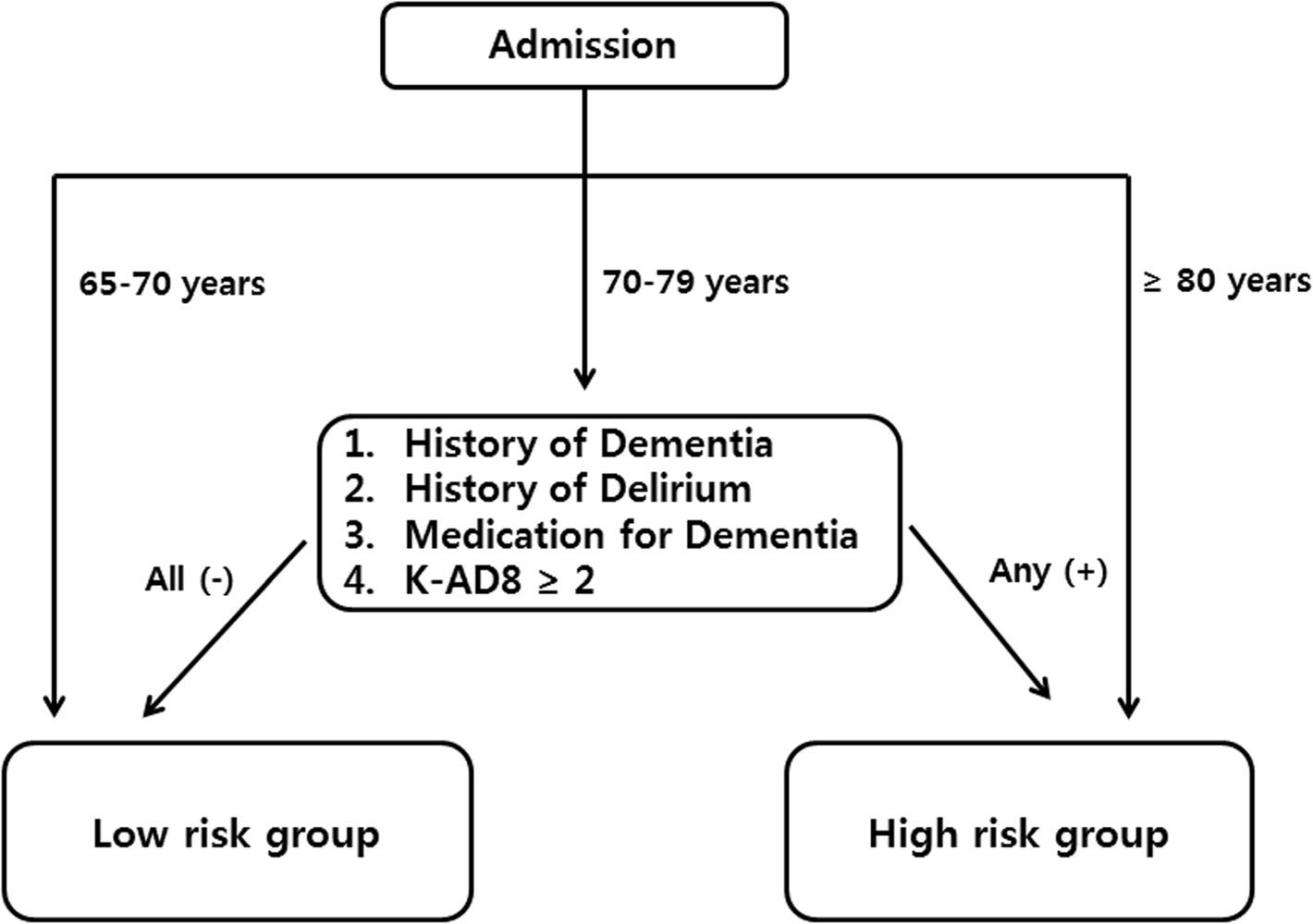
Screening process to identify patients at high risk of delirium. Risk of delirium assessed by age category, history of delirium or dementia, and K-AD8 score

Through an expert team meeting, we developed a screening tool to identify patients who had an increased risk of POD based on reviews of previous studies investigating risk factors for POD [1, 14]. In the screening tool, because the most universal delirium prevention activities are intended to be performed at the age of 70 years and older and because the risk of delirium is known to increase dramatically after 80 years of age, patients aged < 70 years were excluded from screening and were classified as low risk, while patients aged ≥80 years were classified as high risk for POD [1, 5]. Patients aged 70–79 years were classified as high risk for POD if they had a history of dementia or delirium, took dementia medication, or had a Korean version of the AD8 (K-AD8) score ≥ 2, which was identified using a Clinical Dementia Rating over 0.5 (cognitive decline) from 0 (normal) [15] (Fig. 1).

### Implementing a delirium prevention project

**Fig. 2:**
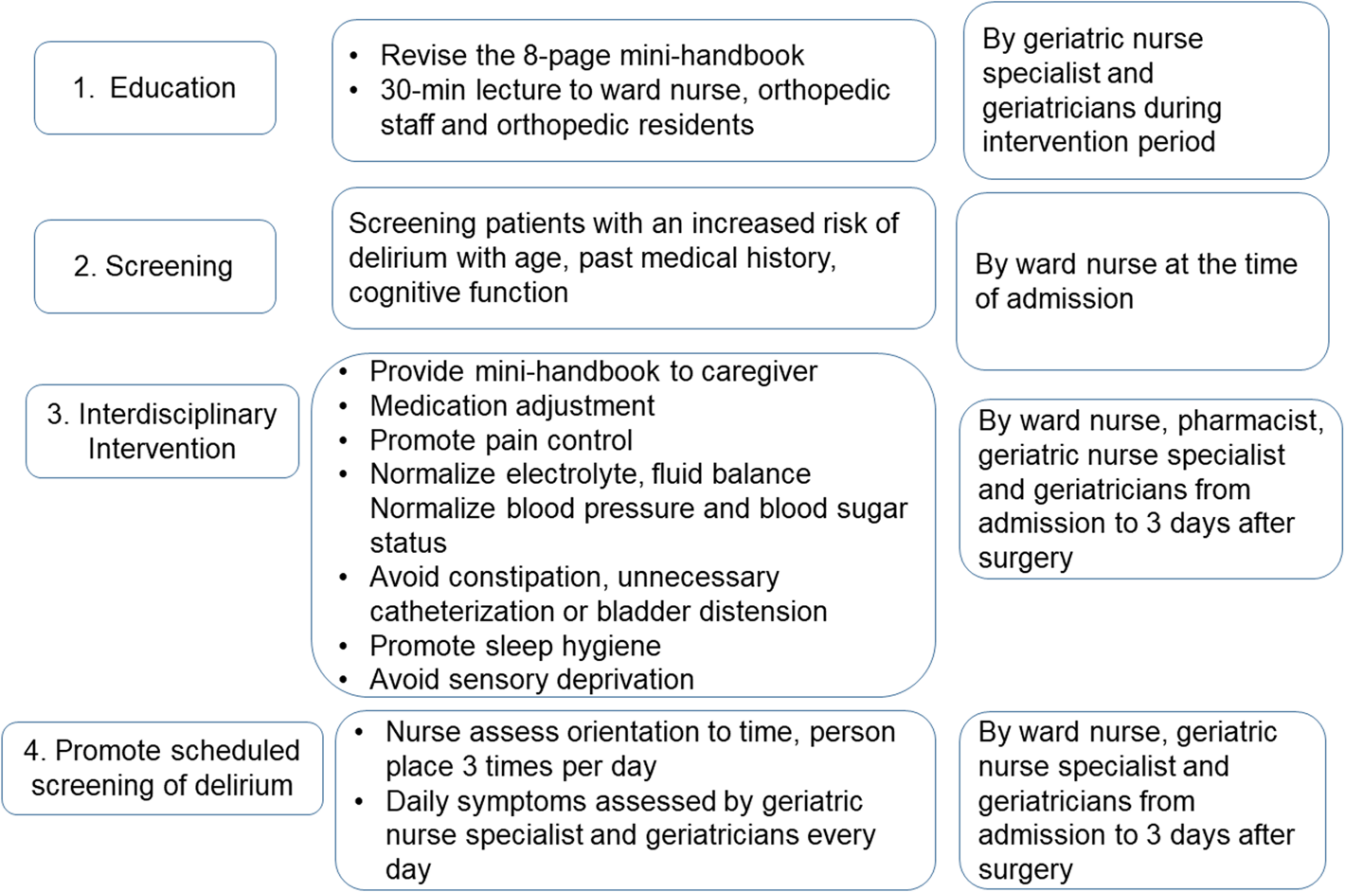
Components of the postoperative delirium prevention project. Multicomponent intervention was conducted including education, screening, interdisciplinary intervention, and promotion of scheduled screening for delirium

Based on a focus group interview, preliminary meetings were held among team members, including geriatricians, a geriatric nurse-specialist, a pharmacist, the senior nurse in charge of the two orthopedic wards, an orthopedic surgeon, and a neurologist. The POD prevention project, comprising four components: education, screening, prevention, and scheduled assessment, was developed (Fig. 2). The first component of the prevention project aimed to enhance education for nurses, orthopedic surgical patients, and their caregivers. We revised an 8-page mini-handbook to include information on delirium (definition, risk factors, symptoms, prevention, prognosis, and Questions and Answers) for patients and caregivers. Nurses working in wards, orthopedic specialists, and residents received a 30-min training session on the pathophysiology, risk factors, prevention, screening, and treatment of POD. The training session was delivered by a geriatric nurse-specialist or by geriatricians. During the pre- and post-training sessions, we evaluated the degree of knowledge regarding POD, previously developed and validated in Korea [16]. Additionally, to maintain ongoing education, training material concerning delirium was added to the education program for new nurses on the team.

The second component of the project aimed to establish a routine screening process to identify patients at risk of POD. We adopted our newly introduced screening tool to be used at the time of admission by a nurse. POD prevention was undertaken in patients identified to be at high risk of POD.

The third component of the project involved the use of an interdisciplinary preventive approach. Nurses provided a mini handbook on POD to patients and their caregivers who had been identified via screening to be at risk of POD. A pharmacist and a group of geriatricians defined a list of 15 medications (chlorpheniramine, diphenhydramine, hydroxyzine, amitriptyline, scopolamine, alprazolam, clonazepam, diazepam, etizolam, lorazepam, triazolam, zolpidem, pethidine, famotidine, and ranitidine) that were reported to potentially cause delirium based on a review of previous studies and the frequency of use of these medications [17, 18]. To make a list of inappropriate medications for the elderly, we used the medication list in our hospital (2012 SNUBH Inappropriate Medication for Elderly Criteria), which is based on the 2008 Screening Tool of Older People’s Prescriptions criteria and 2012 Beers criteria [19]. The medication list for high-risk patients was reviewed by a pharmacist and a geriatrician at admission, and information was provided to orthopedic units by progress notes regarding duplicate prescriptions or medications that could cause delirium or were inappropriate for older adults. Based on this information, alternative medications were suggested by pharmacists and geriatricians. A checklist was provided to high-risk patients detailing the implementation of the evidence-based interdisciplinary approach to prevent POD. The items on the checklist included blood pressure and blood glucose control, pain control, defecation and urination, volume status pre- and post-operatively, electrolyte status, sleep hygiene, and sensory deprivation (the use of glasses and hearing aids), and relevant preventive interventions were performed from the day of admission to 3 days after surgery. Among the check-list items, pain assessment, pain control, and sleep hygiene education were conducted primarily by nurses. Blood pressure, blood glucose, and fluid and electrolyte balance were evaluated and modified by geriatricians. The entire checklist was screened and modified by a geriatrician on a daily basis and was included in the progress notes in the electronic medical record system for ward nurses and orthopedic staff to consider.

The fourth component of the project comprised a scheduled assessment at every nursing staff shift, three times a day, for signs or symptoms of POD. Orientation to time, place, and person was assessed, and if a patient was disorientated, a nurse provided correct orientation (re-orientation). In addition to the ward nurse’s assessment, a geriatric nurse specialist and geriatricians examined the patients daily and evaluated symptoms throughout the day to diagnose POD. If POD was suspected, physicians were encouraged to prescribe atypical antipsychotics when needed or to co-consult with a psychiatrist concerning the patient.

### Outcomes

The primary outcomes were the sensitivity and specificity of the delirium screening tool in predicting POD during the intervention period. The secondary outcomes were the effectiveness of the delirium prevention project in reducing the risk and incidence rate of POD, as diagnosed by a psychiatric consultant or using the Diagnostic and Statistical Manual of Mental Disorders (4th edition) criteria in both cohorts through a retrospective chart review from admission date to discharge date. These diagnostic criteria for delirium were also adapted in the analysis to identify the sensitivity and specificity of the delirium screening tool. To assess the effectiveness of the delirium prevention project, we compared outcomes between the project study period (December 2018 to February 2019) and the same time period 1 year earlier (December 2017 to February 2018). We compared these two phases to assess the effectiveness of the risk reduction of POD and of length of hospital stay. A validation of the delirium screening tool and a comparison of the degree of knowledge of delirium before and after education were performed in the intervention group.

### Statistical analysis

Continuous variables were represented as means (standard deviations [SD]) or medians (interquartile ranges [IQRs]) if the variables were not normally distributed. The paired- or independent t-test and the chi-squared test were used for continuous and categorical variables, respectively. The relationship between the introduction of the POD prevention project, sex, age, or other conventional risk factors and outcomes was determined using logistic regression models. The length of hospital stay was compared using a nonparametric test, the Mann-Whitney U test. Statistical analysis was performed using SPSS version 25.0 (SPSS Inc., Chicago, IL, USA).

## Results

### Performance of the delirium screening tool

**Fig. 3:**
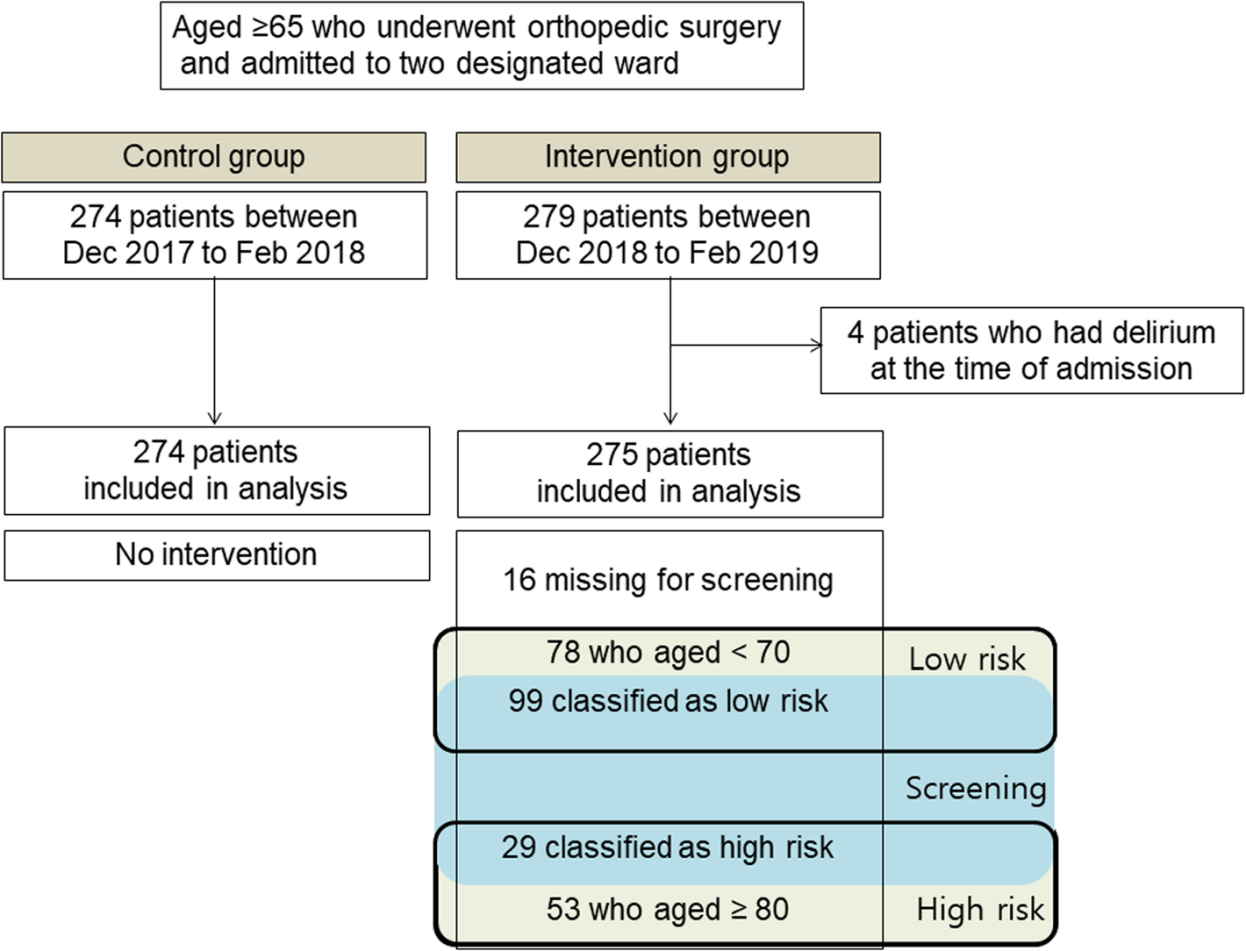
Flow of patients through study. Older adults (aged ≥65 years) who underwent orthopedic surgery and were admitted to two designated wards were recruited, and patients who had delirium at the time of admission were excluded

A total of 275 older adults who were admitted during the study period (intervention group) and 274 older adults who were admitted to the same wards during the same time period 1 year earlier (control group) were included in this study. Baseline patient characteristics such as age, sex, body mass index, and the American Society of Anesthesiologists (ASA) classification were similar between patients in the intervention and non-intervention groups. In addition, confounding factors that could influence the baseline severity of patients, such as the number of medications, number of diseases, existence of dementia medication, education level, or living situation, were not different between two groups (Table 1). Among the 275 intervention group patients, 78 patients were aged < 70 years and 53 were aged ≥80 years. Among the 144 patients (aged 70–79 years) who required screening for delirium/dementia owing to their medical or medication histories and K-AD8 scores at admission, with the exception of 16 patients who missed screening, 99 and 29 patients were classified as low and high risk, respectively. Finally, among the all 275 patients in the intervention group, 82 were classified as being at risk of delirium (Fig. 3).

**Table 1:** Comparison of demographic characteristics of the intervention and non-Intervention groups

	Control (*n* = 274)	Intervention (*n* = 275)	*P* Values
Demographic			
Age (years)	73.6 (6.2)	73.8 (6.3)	0.660
Age ≥ 70	190 (69.3%)	197 (71.6%)	0.556
Age ≥ 80	43 (15.7%)	49 (17.8%)	0.505
Sex (male/female)	78/196	85/190	0.531
Body mass index (kg/m^2^)	25.0 (3.6)	25.6 (5.9)	0.188
ASA class^a^	2.06 (0.6)	2.16 (0.598)	0.050
Number of medications	5.3 (4.1)	5.3 (4.1)	0.953
Existence of dementia medication^b^	7 (2.6%)	6 (2.2%)	0.774
Number of diseases^c^	1.11 (0.79)	1.13 (0.90)	0.728
Type of anesthesia (general/others)	148/126	144/131	0.698
Educational level^d^			
(High/Middle/Low)	58/124/92	52/130/93	0.684
Living situation (home/institutionalization)	272/2	272/3	1.000
Marital status (married/others) ^e^	204/70	197/78	0.457
Operation site			0.301
Spine	74 (27.0%)	67 (24.4%)	
Knee	62 (22.6%)	76 (27.6%)	
Hip	59 (21.5%)	58 (21.1%)	
Shoulder	27 (9.9%)	40 (14.5%)	
Others	52 (19.0%)	34 (12.4%)	
Outcomes			
Hospital stay (days)	9.1 (9.6)	8.0 (8.1)	0.167
In-hospital mortality	5 (1.8%)	1 (0.4%)	0.123
ICU admission	10 (3.6%)	6 (2.2%)	0.325
Discharge site (home/others)	212/57	228/46	0.191

Data are presented as mean (SD) or number (%)

ASA indicates American Society of Anesthesiologists

^a^Data were missing for 71 patients

^b^Dementia medication was defined as donepezil, rivastigmine, galantamine, and memantine

^c^Number of diseases included hypertension, diabetes, heart disease, cancer, and Parkinson’s disease

^d^Educational level was categorized as high (college graduate and higher), middle (middle-school graduate and higher), and low (elementary graduate and lower)

^e^Marital status was categorized as married and others, including single, widowed, divorced, and others

During the study period, with the exception of the 16 patients who missed screening, 82 patients (53 patients aged ≥80 years and 29 patients aged between 70 and 79 years who screened positive) were classified as being at risk of POD and were targeted with individualized delirium prevention strategies. A total of 177 patients (78 patients aged < 70 years and 99 patients aged between 70 and 79 years who screened negative) were classified as being at low risk of POD. Among the patients (*n* = 82) who received the multicomponent interdisciplinary POD prevention intervention and a review of medications in use, 17.1% (*n* = 1 4) and 36.6% (*n* = 30) of patients, respectively, were taking medications inappropriate for older adults or medications reported to potentially cause delirium.

During the course of the delirium prevention QI project, 17 patients experienced POD. Of these, POD occurred in 16 patients who were classified as high risk according to the screening tool. The sensitivity and specificity of the delirium screening tool in estimating POD risk were 94.1 and 72.7%, respectively.

### Effect of delirium prevention project

The incidence rates of POD in the intervention and control groups were 6.2% (*n* = 17) and 10.2% (*n* = 28), respectively. To test the effectiveness of the delirium prevention project in POD prevention, we used multiple logistic regression models to obtain adjusted relative effect measures for the incidence of POD. Variables that could affect the incidence of POD and basic demographic characteristics were adjusted, and the results are shown in Table 2. The POD prevention project showed significant effectiveness with an odds ratio of 0.316 (95% confidence interval [CI]; 0.125–0.800, *p* = 0.015) after adjustment for possible confounders. In the multivariate analysis, the number of medications, existence of dementia medication, type of surgery, and advanced age were also associated with an increased risk of delirium.

**Table 2:** Effect of the delirium prevention project on the incidence of delirium

	Odds ratios	95% CI	*P* Values
Delirium prevention project	0.316	0.125–0.800	0.015
Age (year)	1.220	1.120–1.329	< 0.001
Sex	1.563	0.444–5.506	0.487
Body mass index (kg/m^2^)	0.919	0.802–1.053	0.222
ASA class	0.888	0.377–2.090	0.785
Number of medications	1.315	1.165–1.485	< 0.001
Existence of dementia medication^a^	23.917	3.982–143.638	0.001
Number of diseases^b^	1.151	0.610–2.170	0.664
Type of anesthesia (general vs. others)	0.484	0.132–1.780	0.275
Educational level^c^			0.837
High (Reference)			
Middle	0.653	0.158–2.696	0.555
Low	0.779	0.201–3.022	0.718
Living situation (institutionalization vs. home)	3.553	0.037–338.315	0.585
Marital status^d^ (married vs. others)	1.083	0.395–2.964	0.877
Type of surgery			0.021
Hip (Reference)			
Shoulder	0.788	0.123–5.062	0.802
Spine	0.050	0.006–0.385	0.004
Knee	0.925	0.256–3.344	0.906
Others	0.158	0.027–0.904	0.038

ASA indicates American Society of Anesthesiologists

^a^Dementia medication was defined as donepezil, rivastigmine, galantamine, and memantine

^b^Number of diseases included hypertension, diabetes, heart disease, cancer, and Parkinson’s disease

^c^Educational level was categorized as high (college graduate and higher), middle (middle-school graduate and higher), and low (elementary graduate and lower)

^d^Marital status was categorized as married and others, including single, widowed, divorced, and others

### Effect of medical staff education on knowledge about delirium

The pre- and post-educational training session test scores involving the definition, classification, incidence, risk factors, signs and symptoms, prevention, screening, diagnosis, and treatment of POD were 40.52 and 43.24 out of 49 total points, respectively. The effect of education throughout the study period was analyzed using a paired t-test and was shown to be statistically significant (*p* < 0.001) The median length of hospital stay for the intervention and non-intervention groups was 6.0 days (IQR, 4–9) and 7.0 days (IQR, 4–10), respectively. (Table 1) The delirium prevention project did not shorten the length of hospital stay significantly (*p* = 0.167).

## Discussion

This study demonstrated that a simple delirium screening tool was successful in identifying patients who had an increased risk of POD, with a sensitivity and specificity of 94.1 and 72.7%, respectively. Referring to past systematic reviews utilizing prediction models for POD, we screened a group of patients who were at an elevated risk of POD based on age, dementia, history of delirium, and a simple screening method (K-AD8) for cognitive function. History of dementia, considered to be an irreversible and significant risk factor for delirium, was obtained along with past medical history known by the caregiver and information on medications in use (donepezil, rivastigmine, galantamine, memantine) related to treatment. For screening purposes, rather than evaluating cognitive function in detail, we used an informant-based method that could be performed in a relatively short period of time. The time taken to use the screening tool was approximately 3–5 min, making it simple for nurses to implement during patient assessment upon admission.

Comparing our delirium screening process with the one proposed by Martinez et al., ours classified 82 of 259 (31.7%) patients as high risk, and Martinez’s process classified 201 of 275 (73.1%) patients as high risk [20]. The accuracy performances of both screening process are presented in Table 3. Compared to our screening process, Martinez’s screening process categorized most of the surgical patients as high risk, and all of the patients who experienced delirium were categorized as high risk; thus, their screening process had a very high sensitivity and very low specificity. However, in the high work load setting of a general hospital ward and if there is a shortage of manpower, a screening tool that identifies a relatively tolerable intervention group size is considered preferable.

**Table 3:** Comparison of accuracy parameters for the incidence of delirium between our delirium screening process and Martinez’s screening process

Statistic	Delirium screening process	Martinez’s screening process
Number	259	275
Sensitivity	94.12 (71.31–99.85%)	100% (80.49–100.00%)
Specificity	72.73 (66.65–78.24%)	28.68% (23.21–34.62%)
Positive likelihood ratio	3.45 (2.72–4.38)	1.40 (1.30–1.51)
Negative likelihood ratio	0.08 (0.01–0.54)	0.00
Positive predictive value	19.51% (16.05–23.51%)	8.46% (7.88–9.08%)
Negative predictive value	99.44% (96.33–99.92%)	100%
Accuracy	74.13% (68.35–79.35%)	33.09% (27.56–38.99%)

Data are presented as mean (95% confidential interval)

To our knowledge, this is the first study to evaluate the feasibility and effectiveness of a multicomponent interdisciplinary intervention for POD prevention among hospitalized patients in Korea. Since the reimbursement system for medical treatment or risk management varies in each country, it is not possible to implement a uniform delirium prevention project worldwide. Two geriatricians, one geriatric nurse specialist, one pharmacist, the nurse in charge of the two orthopedic wards, and numerous ward nurses were involved in this 3-month preventive intervention study. The project was feasible to maintain, with team members (geriatricians and geriatric nurse specialists) investing 1 additional hour on average per day and ward nurses paying more attention to patient orientation and checking re-orientation during each shift.

This delirium prevention project may be of help in improving knowledge among medical and nursing staff and in identifying patients at risk of POD. This project showed the possibility of a potentially feasible delirium prevention project that might be beneficial to older patients undergoing orthopedic surgery, with the results showing a significant reduction of 39% (from 10.2 to 6.2%) in the incidence of POD and a > 60% reduction in the risk of POD compared to the control group. Our program exhibited a trend toward a reduction in the length of hospital stay (median of 7 days to 6 days), but this was not statistically significant. The effects of POD prevention may vary according to the intensity or duration of the study intervention and design, and the effects of our project were comparable to those reported in previous studies [21–23]. Furthermore, a variety of factors, including the patient’s condition, presence of surgical drains, home situation, or social resources, all of which can affect length of stay, may have contributed to the lack of significance regarding this outcome.

Problems related to falls, quality of care, and patient safety are of increasing importance; however, the extent to which delirium can be exacerbated in such circumstances with long-term consequences remains unclear. POD may increase the risk of falls, postoperative complications, and functional decline, and POD has been shown to increase the risk of institutionalization [24–26]. Therefore, in an aging society, it is very important to develop and implement a strategy for POD prevention to ensure that aging patients are treated as safely and effectively as possible.

The strength of our study is its novelty in demonstrating the feasibility and effectiveness of an intervention that targeted POD prevention in situations where it was difficult to invest much manpower and expense in delirium prevention. Although this QI project was implemented over a relatively short period of time, it was possible to implement it effectively without having to recruit volunteers or employ additional personnel, and the incidence rate of POD was effectively reduced in the intervention group.

However, our study also had limitations. First, its design was an observational before-after study involving a retrospective review of medical records rather than a prospective randomized controlled trial; therefore, unmeasured confounders may have influenced the outcomes, and an information bias may have been introduced. Hypoactive delirium might have been underdiagnosed, and baseline cognitive function could not be adjusted. However, the incidence of POD in this study was found to be comparable to that reported in a previous meta-analysis involving patients who underwent orthopedic surgery [4]. Second, we conducted this study in one teaching hospital and could not include other important hard outcomes such as short-term (one-year) mortality or injurious fall due to the short observation period. Therefore, it is not possible to determine the generalizability and long-term effect of our findings. Third, delirium was confirmed based on a retrospective chart review; thus, delirium might have been underdiagnosed due to conditions such as hypoactive delirium, overcrowding, or fast workflow, especially in the control cohort. Because delirium was prospectively evaluated up to 3 days after surgery in the intervention group, which is the most frequent period of POD, more delirium could be detected in the intervention group than in the control group. Moreover, the performance of the delirium screening process at admission was assessed in the intervention cohort. These components could lower the specificity of the screening process. Thus, the performances of the screening tool should be interpreted cautiously. Further cohort studies to evaluate the delirium screening process without intervention are warranted. Prospective randomized controlled trials involving multiple institutions and adding new intervention components such as patient mobilization, as well as a longer study duration, are also warranted. Fourth, although we provided education for nurses and physicians, the knowledge test was conducted before and after the education. Therefore, although improvement of the test score may be the effect of education, the confounding effect of the re-test effect cannot be excluded.

## Conclusion

This study reported about a simple screening tool that successfully identified patients at high risk of POD at admission and selected groups requiring further pre-emptive intervention. The POD prevention project was feasible to implement, effective in preventing delirium, and improved knowledge regarding delirium among the medical staff and caregivers.

## Data Availability

The datasets used and analyzed during the current study are available from the corresponding author on reasonable request.

## Abbreviations

ASA: American Society of Anesthesiologists
HELP: Hospital Elder Life Program
IQR: Interquartile range
K-AD8: Korean Version of the AD8
POD: Postoperative delirium
QI: Quality improvement
SD: Standard deviation
